# The Structure of Essentialist Explanations of Necessity

**DOI:** 10.1002/tht3.436

**Published:** 2019-12-02

**Authors:** Michael Wallner

**Affiliations:** ^1^ University of Graz

**Keywords:** essence, necessity, explanation, infinite regress, structure

## Abstract

Fine, Lowe and Hale accept the view that necessity is to be explained by essences: Necessarily *p* iff, and *because*, there is some *x* whose essence ensures that *p*. Hale, however, believes that this strategy is not universally applicable; he argues that the necessity of essentialist truths cannot itself be explained by once again appealing to essentialist truths. As a consequence, Hale holds that there are *basic necessities* that cannot be explained. Thus, Hale style essentialism falls short of what Wilsch calls the *explanation‐challenge* (EC) for the metaphysics of necessity. Without endorsing the EC, I argue that Hale's argument for basic, unexplained necessities fails due to a misunderstanding of the structure of essentialist explanations. Getting clear about the structure of essentialist explanations of necessity leads to a re‐evaluation of crucial circularity‐ and regress‐arguments that have been discussed in the debate about essentialism.

## 1 Introduction

Fine (1994), Lowe (2008, 2012), Hale (2013, 2018) and others argue or accept the view that necessity is to be explained by (or grounded in) essences: Necessarily *p* iff, and *because*, there is some *x* whose essence ensures that *p*. Hale, however, believes that this strategy is not universally applicable. In particular, he argues that the necessity of essentialist truths cannot itself be explained by once again appealing to essentialist truths. It is for this reason that Hale holds that there are *basic necessities* that are not—and *cannot*—be explained. Here is a reconstruction of his argument:

(1) Any essentialist truth must itself be necessary. (E_*x*_ *ϕ*(*x*) → □E_*x*_ *ϕ*(*x*))^1^
(2) The *necessity* of an essentialist truth like E_*x*_ *ϕ*(*x*) cannot be explained by appealing once again to the essence of *x* on pain of vicious circularity.
(3) The *necessity* of an essentialist truth like E_*x*_ *ϕ*(*x*) cannot be explained by appealing to the essence(s) of any other thing(s), for that would

(3.i) undermine the claim that *ϕ*(*x*) is true in virtue of *x*'s essence, and

(3.ii) be viciously regressive.

(4) The *necessity* of an essentialist truth like E_*x*_ *ϕ*(*x*) cannot be explained in any other way.^2^
(5) So, the *necessity* of an essentialist truth like E_*x*_ *ϕ*(*x*) cannot be explained.
(6) Hence, there are basic necessities that cannot be explained.

Interestingly, Hale does not take this to be a shortcoming of essentialist explanations of necessity. Rather he thinks of this as evidence for a *nonreductive* interpretation of essentialism.^3^

The point of the essentialist theory is not […] to provide a reductive explanation of any necessities. It is, rather, to locate a base class of necessities—those which directly reflect the natures of things—in terms of which the remainder may be explained. (Hale 2013, p. 158)

Wilsch, on the other hand, holds that the existence of basic necessities would be a problem for essentialism. On his account, Hale style essentialism would fall short of what he calls the *explanation‐challenge* (EC) for the metaphysics of necessity: “We must accommodate explanations of necessity‐truths through sources and avoid […] unexplained necessity‐truths.” (Wilsch 2017, p. 432)^4^ In this paper, I wish to remain silent on the question of whether basic necessities *should* (at all cost) be avoided. Also, I do not intend to argue that essentialism *can* in principle avoid basic necessities. My goal is more modest. What I wish to show is that Hale's argument for basic necessities fails because it rests on a misunderstanding of the *structure* of essentialist explanations of necessity. In particular, I shall demonstrate how a clarification of this misunderstanding leads to a refutation of premises (2) (§2) and (3) (§§3‐4). Hale's worry that appealing to iterated essentiality claims undermines the initial essentiality claim or renders the explanation of necessity viciously circular or regressive is unfounded. While there might be different reasons to refrain from appealing to iterated essentiality claims or different reasons to accept basic necessities, there is a valuable lesson to be learned from the failure of Hale's argument: Some of the crucial circularity‐ and regress‐arguments that have been championed in the debate about essentialism need to be re‐evaluated (§5).

Two more things need to be said in advance: I will not take issue with premise (1). Hale (2018, p. 127) makes an intuitive case for (1) which is very plausible: “[N]othing could fail to have the essence it has; that is, if *x* is essentially *φ*, it is necessary that it is essentially *φ*. (□_*x*_ *φ*(*x*) → (□□_*x*_ *φ*(*x*)).”^5^

Also, I will grant premise (4). Granting (4) amounts to staying in the essentialist framework, according to which only essences are ever sources of necessity. Hence, the success of Hale's argument for basic necessities is premised on the essentialist claim that only essences can serve as metaphysical sources of necessity.^6^

## 2 Premise (2)

Premise (2) claims that explaining the *necessity* of E_*x*_ *ϕ*(*x*)—that is, explaining □E_*x*_ *ϕ*(*x*)—by the fact that E_*x*_ E_*x*_ *ϕ*(*x*) would be viciously circular. It can easily be shown, however, that this is not the case. Take the following list of necessity‐truths and essentialities:

(7) □*ϕ*(*x*)
(8) E_*x*_ *ϕ*(*x*)
(9) □E_*x*_ *ϕ*(*x*)
(10) E_*x*_ E_*x*_ *ϕ*(*x*)
(11) □E_*x*_ E_*x*_ *ϕ*(*x*)
(12) E_*x*_ E_*x*_ E_*x*_ *ϕ*(*x*)

…

(7) is explained by (8), (9) is explained by (10), (11) is explained by (12), and so on. Note that this is *not a chain* of explanations. It is not claimed that the *explanantia* ((8), (10), (12), …) are themselves explained by a later step.^7^ This *series* of explanations would be circular only if (8) were reducible to (7), or (10) to (9), or (12) to (11), and so on. But the whole point of Fine's, Lowe's and Hale's brand of essentialism is that E_*x*_ *ϕ*(*x*) is *not* reducible to □*ϕ*(*x*), that is: essence does *not* reduce to modality. Thus, there is no circularity involved.

Of course, there is a circular explanation in the vicinity: Suppose someone asked why a specific essentialist truth holds, for example, why containing Socrates as a member lies in the very nature of {Socrates}. This question would prompt a slightly confused reaction and seems to be answerable only by a reiteration of that very essentialist fact: Well, it is just part of what {Socrates} is that it contains Socrates as a member! The fact that there only seems to be a circular explanation of an essentialist fact like “E_*x*_ *ϕ*(*x*)” indicates that, as Glazier (2017, p. 2872) puts it, “we have reached the end of the explanatory road.” It is indeed the case that we hit bedrock once we come to essentialist truths, in the sense that essentialist truths admit of no further explanation.^8^ The point here, however, is that this does not entail that the *necessity* of essentialist truths does not admit of any further explanation. Note that what is at stake in premise (2) is not whether essentialist truths can be explained but whether their necessity can. And while the question *why* {Socrates} *essentially* contains Socrates would make us wonder whether the interlocutor has understood the relevant notions, the question why this essentialist proposition holds *as a matter of necessity* does not seem to prompt the same confused reaction. It rather seems to be a perfectly legitimate question to ask.

I dismiss premise (2) as false, since the mere appeal to E_*x*_ E_*x*_ *ϕ*(*x*) in the explanation of the *necessity* of E_*x*_ *ϕ*(*x*) is not circular. Note, however, that even though there is no structural (or syntactical) problem with this explanation (e.g. no circularity), there might be a *metaphysical* problem. If we take the notion of essence at play here to be the *constitutive essence* of *x*, it is likely that iterated essentiality‐claims like E_*x*_ E_*x*_ *ϕ*(*x*) are simply false. If we take the *constitutive* essence of *x* to express what *x* is *in its most core respects*, it is unlikely that the immediate constitutive essence of *x* contains information *about the constitutive essence of x*.^9^ So even though (2) is wrong, the appeal to the essence of *x* in an explanation of □E_*x*_ *ϕ*(*x*) might be blocked for a different reason than Hale thinks.

## 3 Premise (3.i)

It is difficult to grasp Hale's point in what I call his undermining claim (UND) in (3.i):

(UND) Appealing to the essence of an entity different from *x* in the explanation of the necessity of E_*x*_ *ϕ*(*x*) undermines the claim that E_*x*_ *ϕ*(*x*).

I take it that Hale claims (UND) for he holds the following claim, (BI), to be true:

(BI) The modal status, that is, the necessity of E_*x*_ *ϕ*(*x*) is *“built into”* the relevant essence (i.e. the essence of *x*).

I think Hale believes (UND) to follow from (BI). Appealing to the essence of an entity different from *x* in the explanation of the necessity of E_*x*_ *ϕ*(*x*) undermines the essentialist claim with its *built‐into modal status*. But what exactly does (BI) mean? I think Hale's reasoning is that rather than each essentialist proposition requiring an explanation for its necessity, it should be regarded as part of what essences are that they hold by necessity. Note however, that the second part of the last sentence sounds a lot like an explanation of the necessity of E_*x*_ *ϕ*(*x*). In fact, it sounds like an *essentialist* explanation of the necessity of essentialist propositions. After all, “*it is part of what essences are* that they hold by necessity” might be read as the claim that it is *essential to essences* that essentialist propositions are themselves necessary. On this reading, (BI) does not entail (UND). That is to say (BI) cannot be taken as evidence for the fact that appealing to the essence of an entity different from *x* in the explanation of the necessity of E_*x*_ *ϕ*(*x*) undermines the claim that E_*x*_ *ϕ*(*x*). Rather, (BI) simply contains a pretty straight‐forward, *essentialist* explanation of the necessity of essentialist truths in terms of the *essence of essence*. Given that the initial “*x*” in “E_*x*_ *ϕ*(*x*)” does not stand for essences themselves, the essentialist explanation of the necessity of E_*x*_ *ϕ*(*x*) that is entailed by the built‐into‐claim appeals to the essence of an entity different from *x*. This reading of (BI) not only blocks the inference to (UND), it even provides a counterexample to (UND). So, this reading of (BI) allows me to reject (3.i).

What speaks in favor of my reading of (BI) is that I do not have to assume a *nonessentialist* characterization of *what some x is*. The proponent of what I presume to be Hale's reading of (BI) has to make sense of the question of *what* essences are in nonessentialist terms, thereby assuming extra (presumably primitive) ideology. Note, however, that my reading presupposes that we can make sense of the *essence of essences*. Yet, Lowe (2008, 2012) argues that essences cannot themselves have essences for that would result in a vicious infinite regress. If Lowe is right, (BI) cannot be interpreted in terms of the essence of essences and my case against (3.i) is void. So, I need to resist Lowe's regress‐argument against essences of essences. Luckily, there is a compelling case against Lowe's argument in the recent literature. Spinelli (2018) argues that the regress Lowe fears sets off if we allow for essences to have essences is not a vicious one. In order to see his point, we have to get clear on the theory of infinite regress arguments in general.

Wieland (2014) distinguishes between two different theories of infinite regress arguments: the Paradox Theory and the Failure Theory. According to the Paradox Theory, the crucial premise of an infinite regress argument is shown to have regressive consequences that are paradoxical because they conflict with independent considerations, such that the crucial premise has to be rejected—much like in a *reductio ad absurdum*. Take the following example: The crucial premise that each one of our beliefs depends upon some further belief for being justified has regressive consequences that are paradoxical because they conflict with the independent consideration that there is at least one justified belief. Note that on this account of infinite regress arguments the independent considerations (in this case the assumption that there is at least one justified belief) have to be argued for separately. This is not the case on the Failure Theory. According to the Failure Theory, the purpose of an infinite regress argument is not to refute a proposition (i.e. the crucial premise) like on the Paradox Theory, but rather to show that certain solutions to a problem fail “because they get stuck in a regress of problems that must be solved in order to solve the initial one” (Wieland 2014, p. 8). Take, again, the following example: Suppose the problem you are seeking to solve is to explain how a belief can be justified. The alleged solution would be the claim that each one of our beliefs depends upon some further belief for being justified. Since it is clear that only a belief that is itself justified can justify another belief, the solution to the initial problem fails for you would have to solve infinitely many similar problems prior to the initial one.

Note that regress arguments on the Failure Theory are dialectically stronger than regress arguments on the Paradox Theory. This is because if we take Failure instead of Paradox as criterion for viciousness, we do not need to show that the regressive consequences of the crucial premise are paradoxical or unacceptable for any other reason (Wieland 2014, p. 26). It is because regress arguments on the Failure Theory are in this sense “self‐sufficient” that Spinelli (2018, p. 415) takes it to follow from considerations of charity that whenever it is not clear how a regress argument we are planning to criticize is to be construed, if possible, we should construe it according to the Failure Theory.^10^ So here is Lowe's regress construed according to the Failure Theory.

(L1) If essences are themselves entities with essences, then what metaphysically determines what *x* is, is another entity *y* (the essence of *x*).

(L2) What metaphysically determines *y*, the essence of *x*, is yet another entity, *z*, the essence of the essence of *x*, and so on *ad infinitum*.

(L3)* Failure*: The task of determining what *x* is cannot be completed for such a completion would require *infinitely* many similar tasks to be completed first.

According to Lowe, if essences have themselves essences, the essence of *x* fails to determine what *x* is because infinitely many similar tasks of determination would have to be completed prior to the initial task for it to be achieved. However, Spinelli (2018) makes a convincing case that there is no such failure involved here and that, hence, the ensuing regress is not vicious. His argument depends on what he calls the *Relevance Principle* and Fine's (1995a, 1995b) distinction between *immediate* and *mediate* essence.

*Relevance Principle*: Whatever belongs to the essence of an entity has to be *relevant* to the question of what the entity is (Spinelli 2018, p. 422).

*Immediate Essence*: The immediate essence of an entity *x* only includes what has a direct bearing on the question as to what *x* is (Spinelli 2018, p. 423).

*Mediate Essence*: The immediate essence of an entity in the immediate essence of *x* has only an indirect bearing on what *x* is and is, hence, only in the mediate essence of *x* (Spinelli 2018, p. 423).

Fine (1995a, p. 61) introduces the distinction between immediate and mediate essence with the help of the following example:

(F1) It is essential to {Socrates} to contain Socrates.

(F2) It is essential to Socrates to be a man.

Now, do (F1) and (F2) entail that it is essential to {Socrates} to contain a man? Fine's answer is that on the *mediate* conception of “essential” it does, on the *immediate* it does not. It is clear from this distinction between immediate and mediate essence that the latter but not the former is subject to chaining. Against the background of the *Relevance Principle* we can take the immediate essence of some entity *x* to contain *all* and only those things that are *directly relevant* to *x*.

To see Spinelli's response to Lowe's regress argument against the possibility of essences of essences, it is important to clearly see the crucial task with regard to which there is alleged failure when we allow for essences to themselves have essences. The crucial task is to determine what *x* is essentially. According to *Relevance Principle* and *Immediate Essence*, there is no failure with regard to this task. If, as I think is plausible, we take *y* as the *immediate* essence of *x*, *y* contains all that is directly relevant for *x* and is thus able to determine what *x* is (in this direct sense) without any other task further down the (infinite) list being completed first. This is because the question of what *y*, the essence of *x*, itself is (i.e. the question of the essence of the essence of *x*) has no direct bearing on what *x* is. So, if we are dealing with immediate essences, (L3) is rendered wrong. There is no crucial failure and Lowe's regress is not vicious.^11^

Let us take stock: If Spinelli's response to Lowe's regress argument is sound, there is no structural reason that we cannot make sense of the essence of essence. So, I have what is necessary to advance my reading of (BI) on the ground of which I reject (UND) and (3.i).

## 4 Premise (3.ii)

According to (3.ii) the necessity of E_*x*_ *ϕ*(*x*) cannot be explained by appealing to the essence of *y*, where *y ≠ x* because this would lead into a *vicious regress*. I think that the regress Hale has in mind here is crucially different from Lowe's essence‐regress that I've just discussed. I take it that Hale is concerned with the following issue here: If we explain □E_*x*_ *ϕ*(*x*) by E_*y*_ E_*x*_ *ϕ*(*x*), the latter is an essentialist claim and will, pace (1), be itself necessary. So, we get □E_*y*_ E_*x*_ *ϕ*(*x*), which would in turn be explained by E_*z*_ E_*y*_ E_*x*_ *ϕ*(*x*), which, in turn, would be necessary, … and so on. This means we get the following list of necessity‐truths and essentialist truths:

(13) □E_*x*_ *ϕ*(*x*)
(14) E_*y*_ E_*x*_ *ϕ*(*x*)
(15) □E_*y*_ E_*x*_ *ϕ*(*x*)
(16) E_*z*_ E_*y*_ E_*x*_ *ϕ*(*x*)
(17) □E_*z*_ E_*y*_ E_*x*_ *ϕ*(*x*)

…

(13) is explained by (14), (15) by (16) and so on. It is clear that explaining the necessity of E_*x*_ *ϕ*(*x*) by appeal to the essence of entities different from *x* will give rise to infinitely many necessity‐truths, to infinitely many essentialist truths and to infinitely many essentialist explanations of necessities. The question, however, is whether this really leads into a vicious infinite regress. For reasons, I've outlined in §3, I will, again, base my considerations on the Failure Theory, according to which the viciousness of a regress depends on whether or not there is *explanatory failure*. Note that the crucial task here is to explain the *necessity* of E_*x*_ *ϕ*(*x*) (i.e. to explain (13)). This explanatory task would fail if the explanation would *depend* on the prior completion of *infinitely many* similar explanatory tasks. This, as can be shown however, is not the case. Structurally, the task of explaining (13) is *completed* once we allude to (14) as its *explanans*. Crucially, it is not claimed that (14) itself is explained in anything further down the list. (14) is an essentialist proposition and, hence, it is likely that it does not admit of any further explanation, as I've discussed in §2. It is also important to note that (15) is neither identical with nor reducible to (14). Put differently, explaining the necessity of E_*x*_ *ϕ*(*x*) by alluding to an essence of an entity different from *x* takes the structural form of an infinite *series* of essentialist explanations, *not* that of an infinitely descending *chain* of explanations. Only the latter structure would result in explanatory failure and constitute a vicious infinite regress. There are three things that make it the case that we are not dealing with an explanatory chain here:

- (14) is neither identical with, nor reducible to (15); (16) is neither identical with, nor reducible to (17); and so on.
- It is not claimed that any of the *explanantia* ((14), (16), …) are themselves explained by anything further down on the list.
- (14) suffices to explain (13); that is to say that it is (14), and not (15), that explains (13).

(a) is constitutive of essentialist theories of the Finean, Lowean and Halean brand; (b) stems from the fact that essentialist truths do not admit of any further explanation; and (c) has to do with what Hale calls the *nontransmissiveness* of essentialist explanations of necessity.

Hale holds that whatever the *explanans* of the necessity of *p* is, it must itself be necessary. Yet, it is only in a *transmissive* explanation of the necessity of *p* that the necessity of the *explanans* plays an explanatory role. In a *nontransmissive* explanation of the necessity of *p*, it is merely the truth of the *explanans* that explains the *explanandum*, not its necessity (even though the *explanans* might be indeed necessary).^12^ It can easily be seen that an essentialist explanation of necessity is indeed *nontransmissive*: the necessity of the *explanans*, that is, the necessity of the essentialist proposition does not play an explanatory role. Put differently, even though “E_*x*_ *ϕ*(*x*)” is indeed necessary, it is “E_*x*_ *ϕ*(*x*)” rather than “□E_*x*_ *ϕ*(*x*)” that explains “□*ϕ*(*x*).” It is because of the nontransmissiveness of essentialist explanations of necessity that the task of explaining the necessity of E_*x*_ *ϕ*(*x*) by appeal to an essentialist claim like E_*y*_ E_*x*_ *ϕ*(*x*) is completed at each step. This avoids the chain‐like structure of essentialist explanations of necessity and therefore avoids explanatory failure, which is why the explanatory strategy in premise (3) is not viciously regressive. Again, we can admit that this strategy creates infinitely many essentialist facts and, hence, infinitely many necessities and thereby infinitely many essentialist explanations of necessities. This is hardly problematic, however, since it is likely that we have infinitely many explanations of necessities even on a position like Hale's that allows for unexplained necessities. Even if some necessities are unexplained, it is likely that those that are in need of explanation are still infinitely many. Summing up: the point is that having infinitely many explanations does not entail that any of them is viciously regressive. So, I come to reject (3.ii) as well.

## 5 Conclusion

My goal was to shed light on the structure of essentialist explanations of necessity by criticizing Hale's argument for basic necessities. First, I argued that premise (2) has to be rejected, since explaining the necessity of E_*x*_ *ϕ*(*x*) by appealing once again to the essence of *x* does *not* yield a circular explanatory structure. Taking note of a different reason to doubt the plausibility of iterated essentiality claims like E_*x*_ E_*x*_ *ϕ*(*x*), I moved on to premise (3). (3.i) was dismissed on the grounds of my reading of the claim that the modal status of essence is *built into* the very idea of essence. I cleared the path for this reading by appealing to Spinelli's argument against Lowe's rejection of essences of essences. Concerning (3.ii), I showed that explaining the necessity of E_*x*_ *ϕ*(*x*) by appealing to the essence of an object different from *x* does not yield a viciously regressive explanatory structure. If my criticism is sound, Hale's argument does not succeed in establishing the need for unexplained, basic necessities. None of the premises in Hale's argument provides reason to think that essentialism cannot meet Wilsch's EC. The lesson to be learned concerning the structure of essentialist explanations of necessity is that employing iterated essentiality claims in essentialist explanations of necessity does not *eo ipso* entail a vicious circle or regress. It is, however, an open question as to whether there are different reasons to refrain from appealing to iterated essentiality claims. This is a question for another paper, though.^13^

## Notes

1 Read: If it is true in virtue of the essence of *x* that *ϕ*(*x*), then *necessarily*, it is true in virtue of the essence of *x* that *ϕ*(*x*). Note that I am using “E_*x*_” for the essentiality operator instead of Fine's more popular subscripted box notation “□_*x*_” for the sake of readability.

2 For premises (2)–(4) see Hale (2013, p. 158).

3 For a discussion of a *nonreductive* interpretation of essentialism see Vaidya and Wallner (2018, §7) and Vaidya and Wallner (in press).

4 Clearly, if Hale's argument is sound, essentialism falls short of the second part of the EC, to avoid unexplained necessity‐truths. What about the first part? Hale's nonreductive version of essentialism amounts to modal primitivism. One might think that primitivism will lead to a rejection of the first part of the EC, that is, to a denial that we must accommodate explanations of necessity‐truths through sources. Yet, Wilsch (2017, p. 432) rightly points out that taking modality as a primitive feature of the world does not entail that no modal truth is grounded or explained. That Hale does not subscribe to the latter claim is made clear by him grounding (some) modal truths in essentialist truths, i.e. in their sources.

5 In our notation the formalism would be: E_*x*_ *ϕ*(*x*) → □E_*x*_ *ϕ*(*x*). See also Hale (2013, pp. 132–133). For a recent criticism of this see Mackie (forthcoming), Noonan (2018) and Romero (2019); for a defense against this criticism see Vaidya and Wallner (manuscript). For a more technical argument that any metaphysical source of a necessity must itself be necessary, see Cameron (2010, pp. 93–94) and Hale (2013, pp. 139–140).

6 It is reasonable to restrict this claim to absolute, metaphysical necessity. Whenever I will speak of necessity, I will have this kind of necessity in mind.

7 I will be coming back to this point in §4.

8 This is true at least for grounding‐explanations (Dasgupta 2014) and for what Glazier (2017) calls “essentialist explanations” of essentialist truths. Yet, one way of explaining an essentialist proposition might be by bringing it under a universal generalization. For example, we could answer the question of why it is essential to {Socrates} to contain Socrates by saying that for all *x*, containing *x* lies in the very nature of {*x*}. Thanks to an anonymous reviewer from *Thought* for bringing to my attention this necessary restriction to the claim that essentialist truths cannot themselves be explained.

9 See Dasgupta (2014, p. 591) and Glazier (2017, pp. 2886‐2887) for a discussion of this argument.

10 See Bliss (2013) for a systematic argument for the Failure Theory and for the claim that *explanatory failure* is the best available criterion for viciousness for regresses.

11 Note that this response depends on taking the notion of essence in play here to be the immediate one, which I do. Note that for Fine “[t]he constitutive essence of an object, strictly conceived, is its *immediate* constitutive essence” (Fine 1995b, p. 281; *emphasis added*). For further discussion see Spinelli (2018).

12 See also Cameron (2010, p. 144) for discussion.

13 See, for example, Glazier (2017) for further discussion.
